# Combined application of selected heavy metals and EDTA reduced the growth of *Petunia hybrida* L.

**DOI:** 10.1038/s41598-019-40540-7

**Published:** 2019-03-11

**Authors:** Aqib Hassan Ali Khan, Tayyab Ashfaq Butt, Cyrus Raza Mirza, Sohail Yousaf, Ismat Nawaz, Mazhar Iqbal

**Affiliations:** 1 Department of Environmental Sciences, Faculty of Biological Sciences, Quaid-i-Azam University, 45320 Islamabad, Pakistan; 2 Department of Architectural Engineering, College of Engineering, University of Hail, Hail, Saudi Arabia; 3 Department of Environmental Sciences, Biotechnology Program, COMSATS University Islamabad, Abbottabad Campus, Abbottabad, Pakistan

## Abstract

Up till now, despite of well-developed ornamental market, very little information is available on *Petunia hybrida* L. tolerance against heavy metals (HMs), which can contribute in both beautification of urban dwellings, as well as potential in phytoremediation. Therefore, hydroponic study was conducted to check the effects of Cd, Cr, Cu, Ni and Pb individually (50 and 100 μM) and with co-application of EDTA (2.5 mM) in Hoagland’s nutrient solution. Results indicated higher uptake of Cd, Cr, Ni and Pb in above ground parts, and Cu in roots, further the co-application of EDTA enhanced HMs uptake in *P. hybrida* L. This uptake accompanied changes in biochemical stress indicators, included significantly higher MDA, H_2_O_2_ contents and electrolyte leakage with reduced chlorophyll a, chlorophyll b, total chlorophyll and carotenoid content. Upon exposure to HMs increased antioxidant enzyme activities (CAT, POX, GST, APX, and SOD) were noted. Though selected HMs can be removed by using *P. hybrida* L., the findings of current study indicated that the direct exposure of *P. hybrida* L. to Cd, Cr, Cu, Ni and Pb damaged the plant’s aesthetics, and to use *P. hybrida* L. for beautification of urban landscape or phytoremediation, appropriate soil modification should be included.

## Introduction

With the increase in human population, ecological threats associated with the pollution have also escalated^1,2^. Among the responsible agents that are deteriorating the environment, include the heavy metals (HMs)^3,4^. They are known to accumulate to alarming levels in food chain through the soil contamination^5^. Some of these (like Cd, Pb, and Hg) are not necessary, while others (like Cu, Fe, Mn, Zn, and Mo) act as essential micronutrients for plant growth, and excess concentrations affect plant development as well as wide range of physiological and biochemical processes^6^. Phytoremediation is the use of plants (with or without the associated microorganism) for the treatment of notorious contaminants. It has been widely accepted and applied in last few years. As this methodology is cost effective, sustainable, eco-friendly, non-intrusive, it is expected to play crucial role at industrial scale, if implemented with proper consideration (type of pollutant, seasonal variation, composition of waste generated, and variety of plants used)^7,8^. Though metallophytes, notably hyperaccumulators are attractive option for the removal of HMs, but the associated problems of low biomass production and slow growth as the demerits^1^. In last few decades much of the attention was given to crop plants, while pint-sized information is available for ornamental plants and the effect of HMs on them^9^.

Ornamental plants have distinct advantages over robust crops. Compared to crop plants, they offer reduced risk of bioaccumulation of the HMs in the food chain, if they are found to show hyper/accumulation or tolerance capacities^10^. Their use can also redecorate the aesthetics of the polluted sites at the same time that provide them distinction over other hyper/accumulators^1^. The other scenario that can be developed with ornamental plants is that they will suffer with HMs toxicity. With the improved standards for life style and urban structuring, to a greater extent, ornamental plant can be used in parks, streets and other urban surroundings and there will be an expected increase in different HMs sources such as treated waste water for irrigation, automobile, and industries waste exposure^9^.

To identify the tolerance potential of ornamental plant, a robust and thorough screening is needed. Therefore, an exhaustive screening of potential candidate plants is warranted for any effective application of phytoremediation technology and identification of tolerance limit. *Petunia hybrida* L. is a very commonly grown plant for ornamental purposes around the world and has also been used as a model plant in scientific research work, with well-established agronomic practices for the cultivation^11^. We performed the experiments with *Petunia hybrida* L. in hydroponics and exposed them to Cd, Cu, Cr, Ni, and Pb. These HMs were selected for the study, as they are listed among the priority pollutants under the clean water act and are known for their deleterious impacts on environment, notably plants and animals (including human)^3,5^. Advantage of performing experiment in hydroponic system is that by this way it is possible to identify the actual potential of a plant without the influence of soil and associated microbial population, and to know how these plants will perform with the increased metal bioavailability and mobility. It is well established that Ethylenediaminetetraacetic acid (EDTA) enhanced translocation of HMs in root and shoot^12^, there is no evidence whether the use of this chelating agent can promote or demote the growth of *Petunia hybrida* L., and hence here in this work we investigated the effect of selected HMs (Cd, Cu, Cr, Ni, and Pb) on the plant physiology, biochemical characteristic, enzyme activity and metal uptake potential, with and without EDTA.

## Results

### Growth response

The exposure to selected HMs resulted in reduction and decrease in plant physiological parameters of *P. hybrida*. Studied parameters included total leaves per plant, root and shoot length, leaf area, fresh and dried weights of leaves, root and shoot (Table 1). The use of EDTA in Hoagland’s nutrient solution resulted in significant reduction of studied physiological parameters, when compared with control, and EDTA along with each selected HMs induced significant higher reduction of physiological parameters, in contrast to treatments in which only plants were exposed to HMs without EDTA. Comparing the effect of selected metal on physiological parameter, statistically significant reduction of leaf number in Cu 100 µM + EDTA (~6 leaves per plant), leaf area in both exposure concentrations of Cd, Cu and Cr with EDTA (ranging between 1.34 to 0.81 cm^2^), leaf FW in Cu 100 µM + EDTA (0.63 g), leaf DW in Cd 100 µM + EDTA (0.05 g), root length (cm) and FWs (g) in Cd 100 µM (2.1, and 0.26, respectively), root DW in Ni 100 µM + EDTA (0.03 g), shoot length in Cd 100 µM + EDTA (3.24 cm), shoot FW in Cd, and Cu in 100 µM with EDTA (0.21 and 0.23 g, respectively), and shoot DW in Cd, and Cu 100 µM + EDTA (0.3 in both cases) was observed.

**Table 1:** Physiological parameters of *P. hybrida* L. with reference to HMs and EDTA exposure.

	Total leaves plant^-1^	Leaf Area (cm^2^)	Leaf fresh weight (g)	Leaf dried weight (g)	Root length (cm)	Root Fresh weight (g)	Root dried weight (g)	Shoot length (cm)	Shoot fresh weight (g)	Shoot dried weight (g)
Control^a^	25.00 ± 1.00 a12345	15.25 ± 1.72 a12345	36.55 ± 1.12 a12345	5.24 ± 0.27 a12345	31.39 ± 1.07 a12345	7.96 ± 0.32 a12345	1.01 ± 0.03 a12345	30.29 ± 1.27 a12345	25.21 ± 14.65 a12345	2.71 ± 0.1 a12345
Control + EDTA^a^	19.00 ± 1.00 b12345	4.51 ± 0.23 b2,c13, d45	10.78 ± 6.29 c123, d45	1.24 ± 0.15 c123, d45	15.47 ± 0.98 b13,c245	3.50 ± 0.22 b1345, c2	0.29 ± 0.06 b134, c25	12.86 ± 1.46 b13, c245	10.03 ± 5.79 b1345, c2	1.29 ± 0.15 b12345
Cd 50	14.00 ± 1.00c	7.44 ± 0.98b	18.37 ± 1.01b	1.69 ± 0.15b	11.88 ± 6.86c	1.23 ± 0.04c	0.30 ± 0.01b	6.57 ± 0.35c	2.74 ± 0.18c	0.53 ± 0.05c
Cd 100	13.67 ± 1.53c	5.58 ± 0.66c	8.15 ± 0.24c	0.42 ± 0.05d	2.10 ± 0.40f*	0.26 ± 0.04e*	0.07 ± 0.02d	6.20 ± 0.45cd	0.99 ± 0.18d	0.09 ± 0.02d
Cd 50 + EDTA	9.33 ± 0.58d	1.11 ± 0.15d*	3.34 ± 0.25d	0.42 ± 0.07d	7.74 ± 0.55d	0.57 ± 0.09d	0.19 ± 0.02c	5.06 ± 0.56d	0.96 ± 0.15d	0.08 ± 0.01d
Cd 100 + EDTA	9.00 ± 1.00d	0.81 ± 0.03d*	2.32 ± 0.21d	0.22 ± 0.01d	6.00 ± 0.31e	0.45 ± 0.04de	0.21 ± 0.05c	3.24 ± 0.08e*	0.21 ± 0.04d*	0.03 ± 0.01d*
Cr 50	21.00 ± 1.73b	14.2 ± 1.10a	22.25 ± 1.99b	2.36 ± 0.29b	29.75 ± 17.2b	5.87 ± 0.93b	0.61 ± 0.06b	25.21 ± 14.59b	19.49 ± 1.28c	1.37 ± 0.15b
Cr 100	16.00 ± 1.00c	2.46 ± 0.46c	6.52 ± 1.26d	0.27 ± 0.07d	9.58 ± 0.80d	1.29 ± 0.23d	0.17 ± 0.03de	5.50 ± 0.02e	0.83 ± 0.11f8	0.06 ± 0.01e
Cr 50 + EDTA	19.00 ± 1.00b	1.08 ± 0.03c*	8.64 ± 0.10c	2.36 ± 0.13b	9.75 ± 0.79d	1.54 ± 0.13d	0.21 ± 0.04cd	9.55 ± 0.23d	5.99 ± 0.17d	0.61 ± 0.04c
Cr 100 + EDTA	16.00 ± 1.00c	1.01 ± 0.05c*	6.45 ± 0.34d	0.86 ± 0.06c	7.58 ± 0.41e	0.99 ± 0.03d	0.12 ± 0.02e	5.50 ± 0.49e	3.83 ± 0.88e	0.39 ± 0.10d
Cu 50	13.67 ± 1.53c	6.26 ± 0.08b	13.98 ± 1.23b	1.69 ± 0.15b	14.02 ± 0.10c	1.23 ± 0.04c	0.18 ± 0.03c	5.28 ± 0.42c	2.74 ± 0.18c	0.29 ± 0.04c
Cu 100	9.33 ± 0.58d	4.92 ± 0.56c	8.09 ± 0.21d	0.43 ± 0.09d	11.19 ± 0.47d	0.87 ± 0.10d	0.09 ± 0.02d	5.87 ± 0.29c	0.92 ± 0.05d	0.08 ± 0.02c
Cu 50 + EDTA	10.00 ± 1.00d	1.34 ± 0.04d*	2.32 ± 0.21e	0.16 ± 0.17c	6.00 ± 0.39e	0.75 ± 0.06de	0.12 ± 0.05cd	6.24 ± 0.92c	0.91 ± 0.08d	0.09 ± 0.04c
Cu 100 + EDTA	5.67 ± 0.58e*	1.07 ± 0.04d*	0.63 ± 0.07f*	0.05 ± 0.04e*	5.37 ± 0.28e	0.48 ± 0.04e	0.07 ± 0.02d	5.71 ± 0.03c	0.23 ± 0.07d*	0.03 ± 0.02c*
Ni 50	19.33 ± 1.53b	12.19 ± 7.04b	29.79 ± 1.23b	4.29 ± 0.20b	19.28 ± 11.14b	2.40 ± 0.52c	0.19 ± 0.11b	18.98 ± 1.04b	5.92 ± 0.80c	0.62 ± 0.13c
Ni 100	17.00 ± 2.00b	9.67 ± 0.60c	21.83 ± 1.63c	2.23 ± 0.24c	12.82 ± 7.48d	1.90 ± 0.04d	0.25 ± 0.11b	17.59 ± 1.06b	4.47 ± 0.33d	0.30 ± 0.03d
Ni 50 + EDTA	11.00 ± 1.00c	2.84 ± 0.31e	10.7 ± 0.89d	1.12 ± 0.03d	6.65 ± 0.52e	0.62 ± 0.10e	0.06 ± 0.01c	6.68 ± 0.14d	0.84 ± 0.02e	0.07 ± 0.01e
Ni 100 + EDTA	9.67 ± 0.58c	2.73 ± 0.37e	8.9 ± 0.40e	0.73 ± 0.05e	5.49 ± 0.13e	0.34 ± 0.03e	0.03 ± 0.01c*	6.02 ± 0.52d	0.68 ± 0.03e	0.07 ± 0.01e
Pb 50	22.33 ± 1.53b	11.85 ± 6.84b	28.19 ± 1.29b	4.13 ± 0.64b	20.04 ± 1.57b	2.30 ± 0.41cd	0.35 ± 0.04b	17.6 ± 10.21b	7.35 ± 0.49bc	0.74 ± 0.12bc
Pb 100	17.00 ± 1.00bc	10.00 ± 0.31c	21.83 ± 1.03c	1.94 ± 0.40c	18.02 ± 1.24bc	1.90 ± 0.16de	0.15 ± 0.02c	14.59 ± 8.52c	5.47 ± 0.33c	0.48 ± 0.03cd
Pb 50 + EDTA	16.00 ± 2.00bc	4.34 ± 0.07de	10.70 ± 0.59d	0.98 ± 0.06d	11.32 ± 0.08d	1.83 ± 0.09de	0.09 ± 0.02c	8.84 ± 0.21d	3.89 ± 0.08 cd	0.54 ± 0.01 cd
Pb 100 + EDTA	15.33 ± 0.58c	3.07 ± 0.19e	8.95 ± 1.30e	0.64 ± 0.09d	8.51 ± 0.38e	0.71 ± 0.03e	0.07 ± 0.01c	7.14 ± 0.91d	0.68 ± 0.07d	0.07 ± 0.02e

In each column of metal treatment statistical comparison was done with the same control and control + EDTA. Data was presented in means (n = 3 ± SD).

^a^Alphabets followed by number (1 = Cd, 2 = Cr, 3 = Cu, 4 = Ni, and 5 = Pb) represents statistical differences of control to each of the HMs.

Significantly highest mean was “a” column wise followed by later alphabets for lower means.

Similar small letter in same column within same metal treatment of experiment are non-significant.

*Represents statistically highest reduction among all metal treatments and controls.

**Figure 1:**
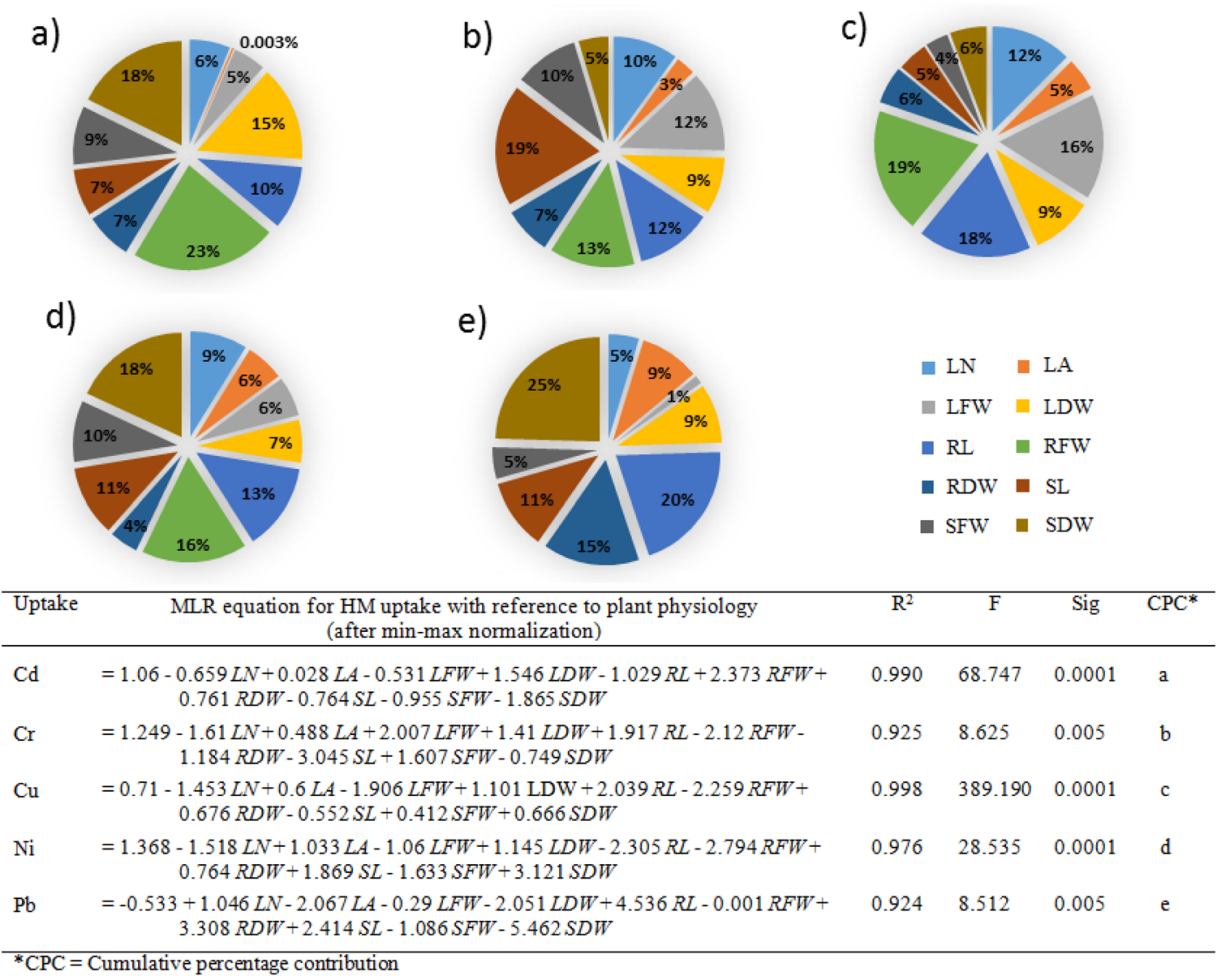
MLR based analysis of HMs uptake and plant physiological parameter of *P. hydrida* L. (including LN = Leaf number, LA = Leaf area, LFW = Leaf fresh weight, LDW = Leaf dried weight, RL = Root length, RFW = Root fresh weight, RDW = Root dried weight, SL = Shoot length, SFW = Shoot fresh weight, SDW = Shoot dried weight, while CPCs for each of the physiological parameters against HM are presented as follow (**a**) Cd, (**b**) Cr, (**c**) Cu, (**d**) Ni, and (**e**) Pb.

Among the studied physiological parameters, significantly high to moderate negative coefficient correlation were noted at *p* = 0.005, where n = 18, between metal uptake and studied parameters, while leaf area, leaf fresh and dried weight were found negatively affected, at the *p* = 0.05 where n = 18, with reference to Pb and Ni. (Supplementary Table S1). The R^2^ value for each of the selected metal uptake model was higher than 0.9, indicating the good fit of the model (Fig. 1). In case of Cd, and Cu the CPC was highest for the root FW (23, and 19%, respectively), shoot length in Cr (19%), while for Ni and Pb the highest CPC was noted for shoot dried wright (18, and 25%, respectively).

### Stress injury

**Figure 2:**
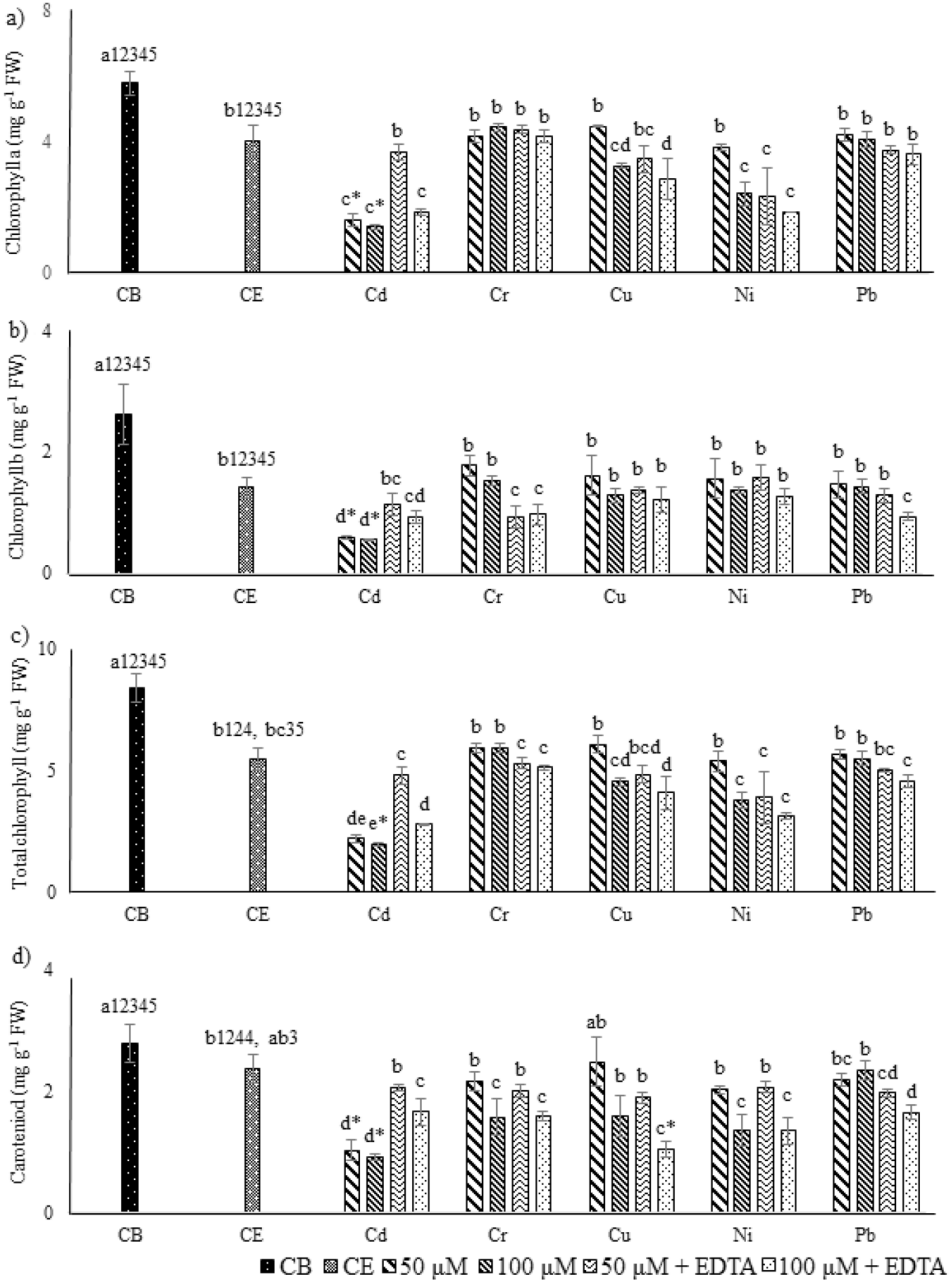
Effects of HMs and EDTA co-application on Chlorophyll and carotenoid contents of *P. hydrida* L. (**a**) Chlorophyll a, (**b**) Chlorophyll b, (**c**) Total chlorophyll, and (**d)** Carotenoid content. Statistical comparison is presented in different series of bars (50 = 50 µM of HM, 100 = 100 µM of HM, 50 + ETDA = 50 µM  + 2.5 mM EDTA, 100 + EDTA = 100 µM  + 2.5 mM EDTA) by using the same control (CB) and control + EDTA (CE). Data are in means (n = 3 ± SD). Alphabets represent statistical differences, number followed by alphabets in CB and CE represents difference with metals (1 = Cd, 2 = Cr, 3 = Cu, 4 = Ni, and 5 = Pb), significantly highest mean was “a” in each series of bars followed by later alphabets for lower means. *Represents significantly highest reduction.

**Figure 3:**
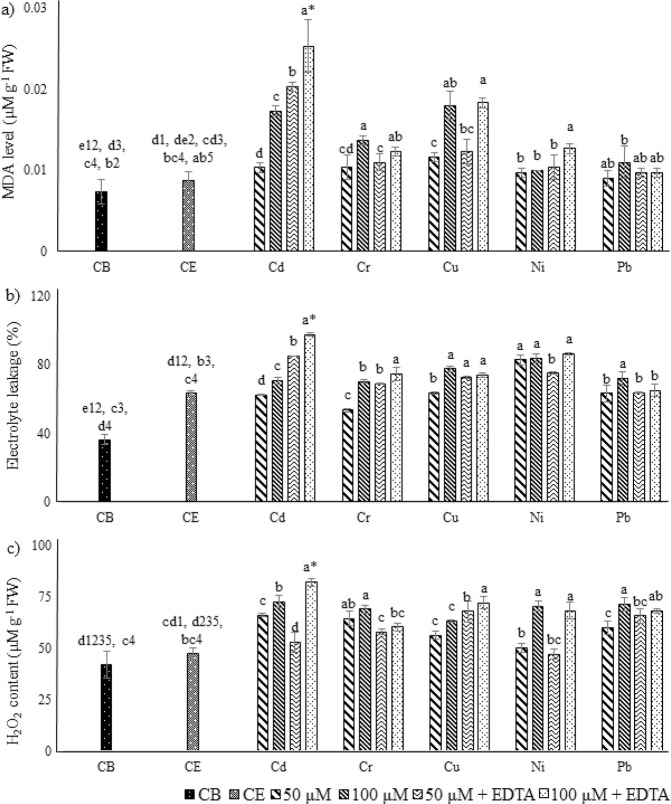
Effects of HMs and EDTA co-application on (**a**) MDA level. (**b**) Electrolyte content, and (**c**) H_2_O_2_ content of *P. hydrida* L. Statistical comparison is presented in different series of bars (50 = 50 µM of HM, 100 = 100 µM of HM, 50+ETDA = 50 µM + 2.5 mM EDTA, 100 + EDTA = 100 µM + 2.5 mM EDTA) by using the same control (CB) and control + EDTA (CE). Data are in means (n = 3 ± SD). Alphabets represent statistical differences, number followed by alphabets in CB and CE represents difference with metals (1 = Cd, 2 = Cr, 3 = Cu, 4 = Ni, and 5 = Pb), significantly highest mean was “a” in each series of bars followed by later alphabets for lower means. *Represents significantly highest reduction.

**Figure 4:**
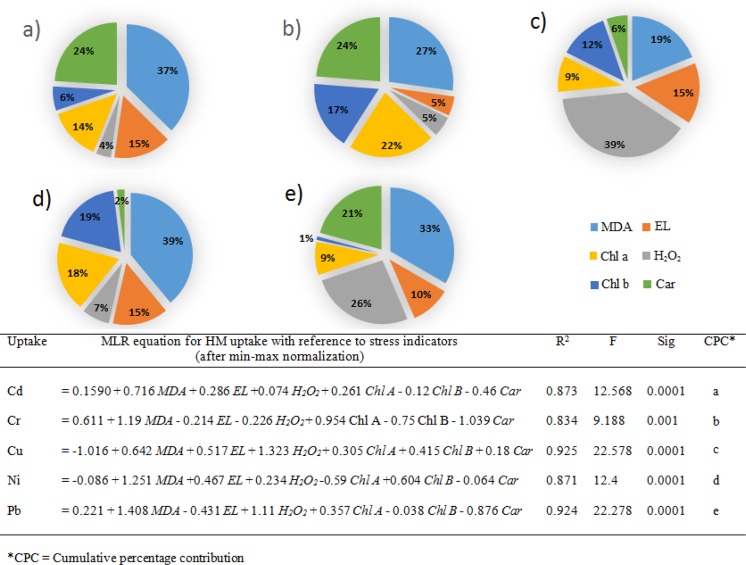
MLR based analysis of HMs stress indicators of *P. hydrida* L. (including MDA = Malondialdehyde content, EL = Electrolyte leakage, Chl a = Chlorophyll a, Chl b = Chlorophyll b, Car = Carotenoid, and H_2_O_2_ = Hydrogen peroxide content), while CPCs for each of the stress indicating parameters against HM are presented as follows (**a)** Cd, (**b)** Cr, (**c)** Cu, (**d)** Ni, and (**e)** Pb.

Effects of selected HMs application on chlorophyll content (chlorophyll per g^−1^ of FW), including chlorophyll a (Chl a), chlorophyll b (Chl b), total chlorophyll (Chl T) and carotenoid (Car) content were presented in Fig. 2. Abrupt decline in Chl a (74.5%), Chl b (79%) and Chl T (76.8%) were noted for Cd treatments, and significantly highest in all cases of 100 µM, when compared with control. For Car, statistically significant decline was noted for Cd treatment at 50 and 100 µM, and with Cu at 100 µM + EDTA (~67%), in comparison to control. The addition of EDTA significantly improved plant growth, in the case of Cd. While, no variation for Cr and Ni was noted. Significantly highest lipid peroxidation (µM of MDA g^−1^ of FW), EL, and H_2_O_2_ were noted for 100 µM Cd treatment with EDTA (Fig. 3). In case of Cr and Pb, highest MDA contents were noted at 100 µM metal concentration (0.013 and 0.011, respectively), while for Cd, Cu and Ni, 100 µM metal treatment with EDTA resulted in highest MDA contents (0.025, 0.018 and 0.012 µM g^−1^ FW, respectively). The highest percentages for EL were noted for Cd, Cr, Cu and Ni, at 100 µM metal treatments with EDTA, with the following trend, Cd (97.7%) > Ni (86.7%) > Cr (74.7%) > Cu (74%). In case of Pb statistically significantly higher EL was recorded for 100 µM Pb concentration (72%), as compared to control. The H_2_O_2_ contents for Cd and Cu were highest, and for Cd and Pb, were also higher with 100 µM metal treatments with EDTA, while in case of Ni at 100 µM metal treatments, in both conditions with and without EDTA, were resulted significantly highest H_2_O_2_ content. Supplementary Table S2, represented the coefficient correlation between selected HMs and stress indicator. MDA content, EL and H_2_O_2_ contents were found strongly positively correlated, while were found negatively correlated for Chl a, Chl b, Chl T and Car with metal uptake. The coefficient correlations were high to moderate between each case. MLR equations and cumulative percentage contributions were presented for metal uptake and studied stress indicators (Fig. 4). MDA contents was found to have highest percentage contribution (39, 37, 33, and 27%, respectively) for Ni, Cd, Pb and Cr. While, for Cu the H_2_O_2_ content resulted in highest CPC (39%). The high MLR coefficients (R^2^) were observed for each metal ranging from 0.873 to 0.925.

### Antioxidant enzyme activity

**Figure 5:**
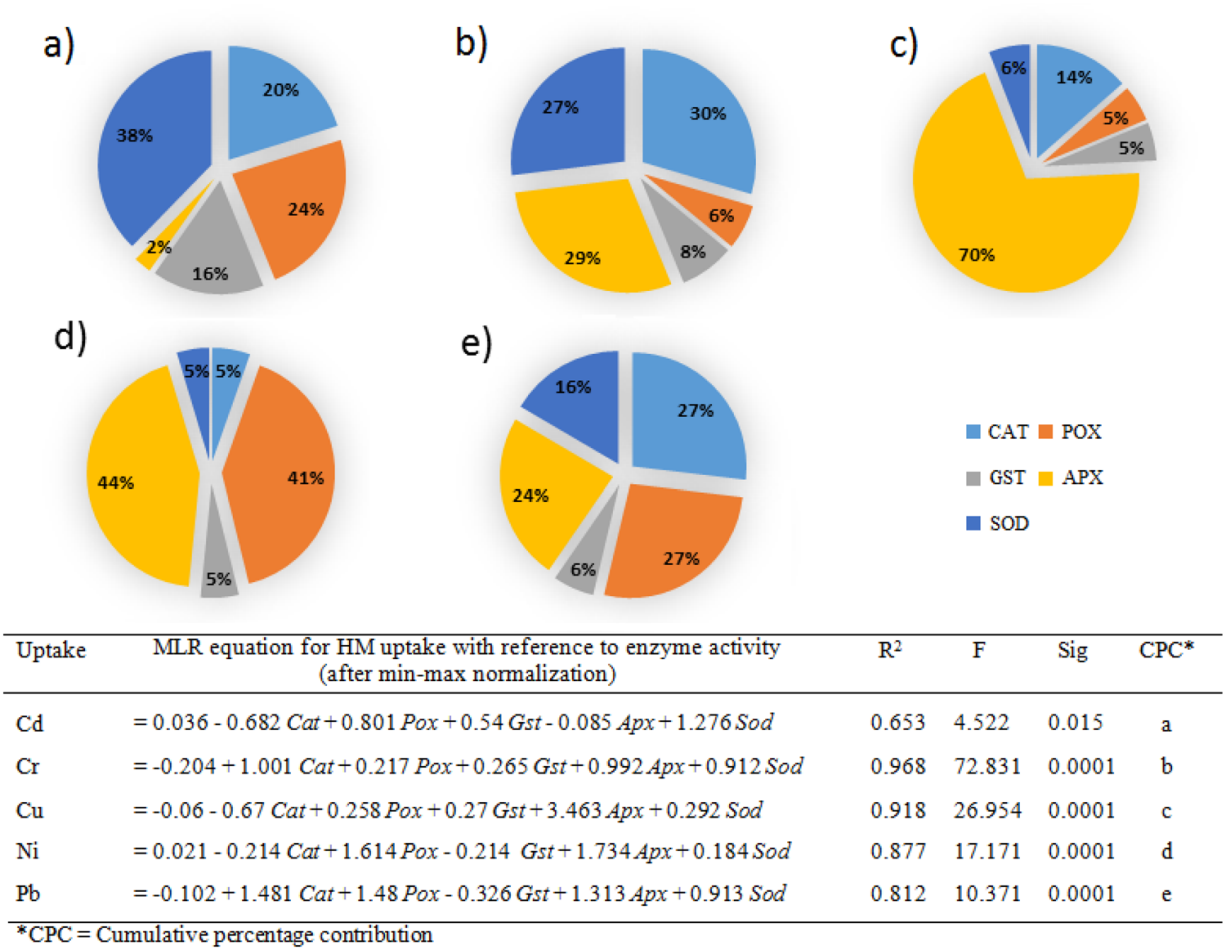
MLR based analysis of HMs effect on enzyme activity of *P. hydrida* L. (including CAT = Catalase, POX = Guaiacol peroxidase, APX = Ascorbate peroxidase, GST = Glutathione-s-transferase, and SOD = Superoxide Dismutase, while CPCs for each of the enzyme activity profile against HM are presented as follows (**a)** Cd, (**b)** Cr, (**c)** Cu, (**d)** Ni, and (**e)** Pb.

The activities of CAT, POX, GST, APX, and SOD content in leaves of *P. hybrida* exposed to Cd, Cr, Cu, Ni and Pb with or without EDTA were presented in Table 2. Enzyme activities (including CAT, POX, GST, APX and SOD) increased with increasing the concentrations of selected HMs from 0 to 50 and 100 µM. The addition of EDTA with HMs, resulted in statistically significant amplification in all enzyme activities of *P. hybrida*, except in the case of POX activity, where highest value was noted for Cr 100 µM (0.88 U g^−1^ of FW), and reduction in POX activity was noted with the addition of EDTA along with Cr at 50 and 100 µM (0.31 and 0.41 U g^−1^ of FW). While, in the case of SOD statistically insignificant variations were noted with or without EDTA at both concentration of 50 and 100 µM with Pb. Upon paralleling the effect on enzyme activities among selected metals, statistically significant increase in CAT in Cd 100 µM + EDTA (1.13 U g^−1^ of FW), POX and GST in Cu 100 µM + EDTA (0.88 U g^−1^ of FW and 0.62 μM min^−1^ g^−1^ FW), APX in treatments of Cd and Ni at 100 µM with EDTA (0.38 and 0.37 U g^−1^ of FW), and SOD in Cd 100 µM + EDTA (3.19 U g^−1^ of FW) were observed. Correlation between enzyme activities and heavy metal exposure was presented in Supplementary Table S3. Among selected enzymes Cd exposure showed strong positive correlation in case of SOD (0.746), while for CAT, POX, GST moderate positive correlations (0.640, 0.613 and 0.633) were noted at the *p* = 0.005, when n = 18, with insignificant correlation for APX. With Cr, strong positive correlations were identified for CAT, GST and APX (0.889, 0.812, and 0.921, respectively), while for POX and SOD activity moderate correlations were observed (0.69 and 0.59, receptively) at the *p* = 0.005 when n = 18. Exposure of *P. hydrida* to copper displayed strong positive correlations with CAT, APX, and SOD (0.733, 0.091, and 0.746, respectively), moderate positive with GST (0.602) at the *p* = 0.005 when n = 18, while weak positive with POX (0.453) at the *p* = 0.05 when n = 18. The treatment with Ni showed strong positive correlations with all enzyme activities at the *p* = 0.005 where n = 18. Furthermore, Pb exposure found to have strong positive correlations with CAT and POX (0.810, and 0.728, respectively), while moderate positive correlation with GST (0.595) and SOD (0.668) at the *p* = 0.005 when n = 18. APX was also found to have moderate positive correlation with Pb exposure (0.530 at the *p* = 0.05 when n = 18). Figure 5 represented the cumulative percentage contribution and MLR equation for each selected metal and studied enzyme activities. In case of Cr, Cu, Ni and Pb significantly higher MLR coefficients (R^2^) were observed. While, with Cd the model’s R^2^ value was found to be moderate. Highest CPC noted for uptake of Cd was with SOD (38%), for Cr with CAT (30%), Cu and Ni for APX (70 and 44%, respectively), and for Pb with CAT and POX (27, and 27%, respectively).

**Table 2:** Effect on enzyme activities of *P. hybrida* L. upon exposure of HMs and EDTA.

	Catalase^+^	Guaiacol peroxidase^+^	Glutathione S-transferase^+^	Ascorbate peroxidase^+^	Superoxide dismutase^+^
Control^a^	0.53 ± 0.06 c12345	0.14 ± 0.02 c15,d234	0.33 ± 0.01 c125, d3, e4	0.03 ± 0.00 c14,d235	1.39 ± 0.07 c125,d3,b4
Control + EDTA^a^	0.54 ± 0.04 c12345	0.17 ± 0.02 c15,d234	0.41 ± 0.02 b1235, c4	0.05 ± 0.01 c145,d23	1.44 ± 0.09 c125,d3,b4
Cd 50	0.56 ± 0.04c	0.17 ± 0.01c	0.34 ± 0.01c	0.05 ± 0.00c	1.65 ± 0.07bc
Cd 100	0.66 ± 0.01b	0.49 ± 0.03a	0.41 ± 0.04b	0.11 ± 0.01b	1.88 ± 0.05bc
Cd 50 + EDTA	0.55 ± 0.1c	0.30 ± 0.03b	0.43 ± 0.02b	0.1 ± 0.01b	1.94 ± 0.03b
Cd 100 + EDTA	1.13 ± 0.05a*	0.46 ± 0.01a	0.49 ± 0.01a	0.38 ± 0.05a*	3.19 ± 0.68a*
Cr 50	0.56 ± 0.03c	0.41 ± 0.06b	0.38 ± 0.02c	0.07 ± 0.01 cd	1.64 ± 0.01b
Cr 100	0.60 ± 0.02c	0.5 ± 0.06a	0.42 ± 0.06b	0.11 ± 0.04bc	1.72 ± 0.11b
Cr 50 + EDTA	0.68 ± 0.03b	0.31 ± 0.07c	0.48 ± 0.28ab	0.13 ± 0.02b	1.67 ± 0.15b
Cr 100 + EDTA	0.8 ± 0.03a	0.41 ± 0.02b	0.53 ± 0.04a	0.28 ± 0.05a	2.11 ± 0.16a
Cu 50	0.57 ± 0.01bc	0.18 ± 0.02	0.34 ± 0.02d	0.12 ± 0.01c	1.86 ± 0.08b
Cu 100	0.75 ± 0.05a	0.45 ± 0.05b	0.47 ± 0.02b	0.16 ± 0.00b	2.23 ± 0.08a
Cu 50 + EDTA	0.62 ± 0.06b	0.37 ± 0.03c	0.46 ± 0.02b	0.14 ± 0.03bc	1.63 ± 0.10c
Cu 100 + EDTA	0.73 ± 0.00a	0.88 ± 0.03a*	0.62 ± 0.02a*	0.26 ± 0.03a	2.01 ± 0.20b
Ni 50	0.63 ± 0.01b	0.28 ± 0.06c	0.37 ± 0.03d	0.03 ± 0.00c	1.49 ± 0.02b
Ni 100	0.64 ± 0.01b	0.47 ± 0.02a	0.4 ± 0.01 cd	0.04 ± 0.01c	1.54 ± 0.13b
Ni 50 + EDTA	0.56 ± 0.03c	0.26 ± 0.03c	0.47 ± 0.01b	0.21 ± 0.04b	1.65 ± 0.12ab
Ni 100 + EDTA	0.84 ± 0.05a	0.36 ± 0.04b	0.52 ± 0.01a	0.37 ± 0.02a*	1.88 ± 0.09a
Pb 50	0.55 ± 0.02c	0.15 ± 0.02c	0.34 ± 0.01c	0.04 ± 0.01c	1.56 ± 0.34ab
Pb 100	0.68 ± 0.04b	0.18 ± 0.01c	0.39 ± 0.03b	0.04 ± 0.00c	1.79 ± 0.21a
Pb 50 + EDTA	0.64 ± 0.03b	0.26 ± 0.02b	0.39 ± 0.01b	0.05 ± 0.01b	1.54 ± 0.07ab
Pb 100 + EDTA	0.73 ± 0.01a	0.38 ± 0.04a	0.49 ± 0.03a	0.07 ± 0.00a	1.80 ± 0.17a

In each column of metal treatment, statistical comparison was done with the same control and control + EDTA. Data was presented in enzyme unit per mg of fresh weight. Data are in means (n = 3 ± SD).

^a^Alphabets followed by number (1 = Cd, 2 = Cr, 3 = Cu, 4 = Ni, and 5 = Pb) represents statistical differences of control to each of the HMs.

Significantly highest mean was “a” column wise followed by later alphabets for lower means.

Similar small letter in same column within same metal treatment of experiment are non-significant.

*Represents statistically highest mean among all metal treatments and controls.

^+^All enzyme activities are expressed in U g^−1^ FW of plant, while for GST is in μM min^−1^ g^−1^ FW of plant.

### Heavy metal uptake

**Figure 6:**
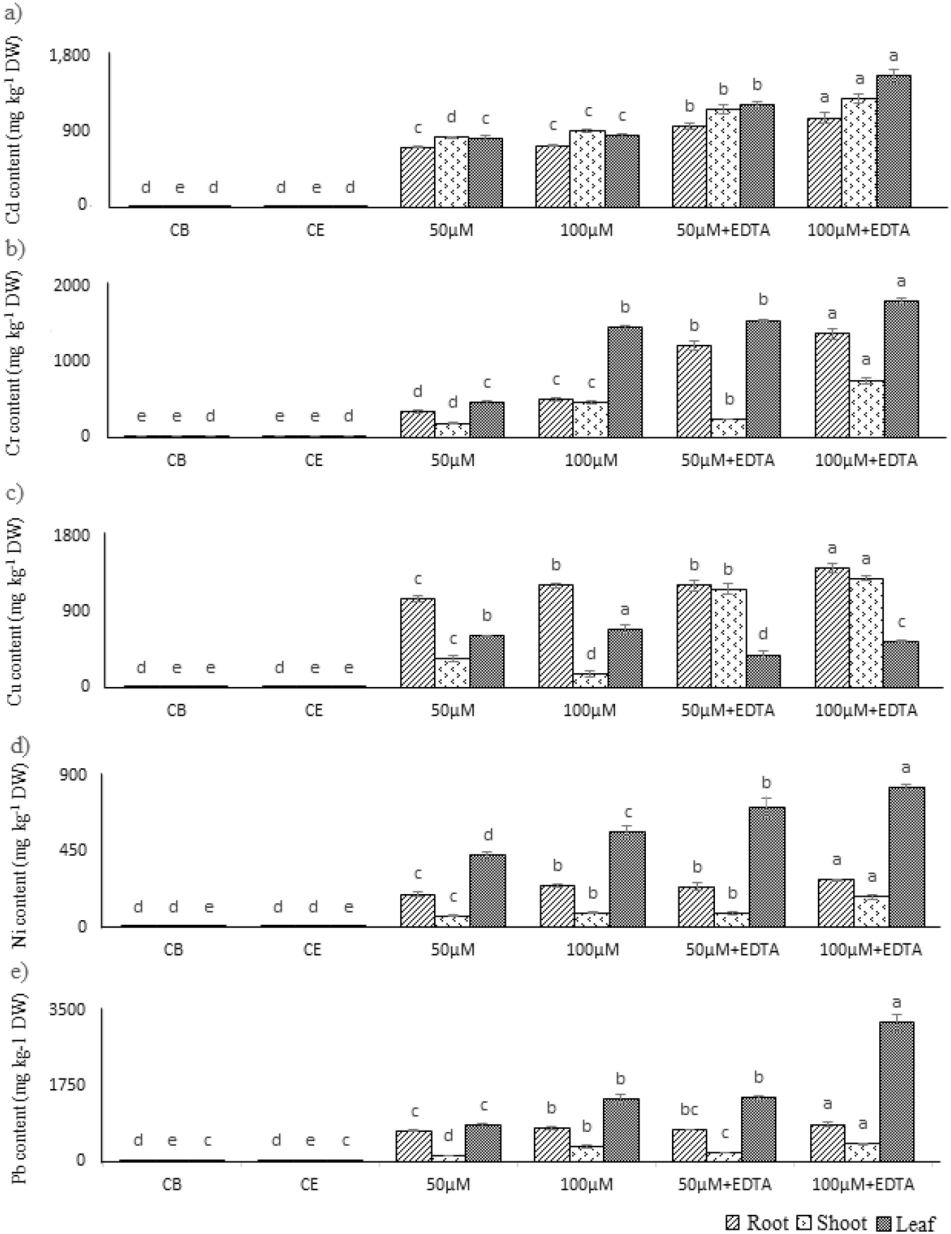
Uptake and compartmentalization of HMs in *Petunia hybrida* L. Statistical comparison is presented in different series of bars (Root, shoot, and leaf of CB, CE with (**a)** Cd, (**b)** Cr, (**c)** Cu, (**d)** Ni, and (**e)** Pb). Data are in means (n = 3 ± SD). Alphabets represents statistical differences; significantly highest mean is “a” in each bar series followed by later alphabets for lower means.

Heavy metal uptake by *Petunia hybrida* L. was dose dependent, as increase in metal concentration resulted in increased HM content with in the plant tissues (Fig. 6). The use of EDTA also exacerbated the metal content uptake. However, the pattern of compartmentalization, i.e. metal content in each of the plant compartment (root, shoot and leaf), varied. In case of Cd, the distribution of metal was found comparatively uniform, but with the addition of EDTA higher Cd content was noted in leaf, followed by shoot and root. Leaf contained highest metal content, when plants were treated with Cr, followed by a dose dependent increase in shoot, while in root the content of Cr was comparatively uniform with no statistical difference. Application of EDTA with Cr resulted in highest content of Cr with in the leaf, followed by root and then shoot. Copper content was found highest in root in all the treatments, interestingly with co-application of EDTA, the shoot metal content increased significantly. With Ni and Pb, the metal uptake was found to be highest in leaf and is influenced by both the dose of application and addition of EDTA with metal treatments.

## Discussion

Use of ornamental plant is expected to grow with the passage of time, not just because they can improve the aesthetics of the urban dwellings and medicinal advantages, but also in the field of phytoremediation, as this discipline is an ever evolving method and much attention was not given on the ornamental plants^9,10,13^. Therefore, it will be a good approach to investigate commonly used ornamental plants. This will not only help to identify the potential for any possible use in phytoremediation technology, but will also in the scaling the tolerance limit against HMs, assistance to maintain the vibrant aesthetics of these plants when used in urban surroundings (like roads, gardens, and walking lanes) under the expected stress of environmental contaminants spread due to anthropogenic activities (vehicular exhaust, irrigation with contaminated water, soil erosion, and even dust scattering due to pedestrian). This study was designed to understand the tolerance level and metal uptake pattern of *Petunia hybrida* L., when exposed to cadmium, chromium, copper, nickel and lead, with and without addition of EDTA, and to best of our knowledge no such competitive study was done. The use of EDTA for ameliorated extraction of heavy metals was well documented in already published data with other plants like *Brassica napus*^4,12^, *Brassica rapa*^14^*, Phaseolus vulgaris* and *Zea mays*^15^, and *Helianthus annuus*^13,16^. The effect of addition of EDTA was also found to vary (positive or negative) among different plants^4,13,14^.

### Effects of HMs and EDTA exposure on plant physiological status

In the present study, it was observed that addition of EDTA enhanced the HMs uptake, but enhanced metal uptake resulted in reduced plant growth. The studied physiological parameters of *Petunia hybrida* L. were negatively influenced by the increasing the concentration of selected HMs in the hydroponic media (Table 1 and Supplementary Table S1). The impact was further escalated, when the HMs were provided with co-application of EDTA. The negative impact of HMs was dose dependent, while for EDTA the negative impact was due to increased toxic HMs uptake and the hindrance in essential metal uptake as EDTA bind non-specially with metals, resulting in chelation of metals required by plant along with the toxic HMs^16^. Further the chelator mediated toxicity also reduced the plant biomass, limiting the metal uptake^17^, as with low biomass limited HM is expected to be translocate. It should further be noted that a higher HM concentration in plant tissue by the addition of metal chelating agent did not necessarily mean a better removal efficiency, as the change in biomass and other physiological parameter are the determining factors^16^. This can be better understood by identifying the contribution of each parameter towards HM uptake. Cumulative percentage contribution of each of the physiological parameters against total metal uptake (Fig. 1) was performed and checked how metal uptake is affected by reduction or increase in each of studied parameter. For Cd, and Cu the highest CPC was for the root FW (23, and 19%, respectively), indicating that root fresh weight was significantly reduced by the uptake of these metals (Supplementary Table S1). Root fresh weight and Cu and Cd uptake were negatively co-related. Shoot length the physiological parameter is most affected. Ivanov, *et al*.^18^ performed root toxicity assay with *Zea mays* L. and proposed that metal toxicity was declined as follow Cu ≈ Ti > Ag > Cd > Hg > Co > Zn > Pb, and the results were in accordance to our *Petunia hybrida* L. HMs exposure experimentations. When *Petunia hybrida* L. plants were exposed to Cr, Ni and Pb highest CPC (19, 18, and 25%, respectively), was noted with shoot length for Cr and shoot dried weights for Ni and Pb, suggesting that negative influence on shoot occurred upon exposure of these HMs, and this was also evident by seeing the coefficient co-relation (Supplementary Table S1).

### Plant biochemical and enzymatic status with reference to HMs and EDTA stress

Upon exposure to HMs, plants are expected to undergo stressed condition, contributing to metal uptake that leads to generation of reactive oxygen species (ROS), reduced plant turgidity, lipid peroxidation and decreased chlorophyll content^19^. Multiple toxic effects occur upon exposure to HMs, primarily due to formation of ROS, inhibiting cellular processes at different levels. The instability of ROS contributes; (a) damage cellular components, and (b) an important tributary for the induction of defence system^20^. In the present study reduced (negative correlation) in Chl a, Chl b, Chl T, and Car, while increased (positive correlation) MDA, H_2_O_2_ contents and EL were recorded upon the stress induced by each selected HM at 50 and 100 µM (Figs 2 and 3, Supplementary Table S2). The addition of EDTA resulted in improvement of plant pigments in case of Cd, reduction with Cr, Cu and Ni, while no significant changes in Pb. The MDA content represents damage due to lipid peroxidation, electrolyte leakage is a good indicator to check the integrity of plant cell membrane, while higher H_2_O_2_ content shows the production of ROS^12,21^. Upon exposure to HMs, and successive increase in the exposure concentrations led to increment in EL, MDA, and H_2_O_2_ content, while the addition of EDTA did not showed any positive or negative impact on these parameters, except for Cd, where significantly higher EL, MDA, and H_2_O_2_ contents were noted. Hence, it can be said that the HM induced stress to *Petunia hybrida* L. was not reduced upon addition of EDTA, despite it was known for the metal chelating activities^4,12,17^. The effects on plant that occur due to EDTA facilitated HMs uptake vary broadly based on HM, and the plant effected^4,12^. The lipid peroxidation and oxidative damage due to metal uptake is detrimental, and in this work MDA content (for Cd, Cu, Ni and Pb) and H_2_O_2_ (for Cu) were found to have highest CPC, for metal uptake model. It is inferred that upon exposure to HMs, the growth of *Petunia hybrida* L. plant was decrease due to ROS formation and lipid damage due to peroxidation. If there are no other options for the irrigation of *Petunia hybrida* L. except with HMs contaminated water, appropriate measures should be taken. These options include soil conditioners (individually or in combination) like, biochar, biosolids, compost, organic acids, and plant growth promoting bacterial amendments, as they have proven to reduce the toxic impacts of HMs on plants, which were not found achievable by using synthetic chelator like EDTA^17,22,23^, and in the current investigation as well.

The stress due to oxidative damage, induced by HMs uptake, is tackled plants with the help of defence mechanisms against oxidants. One of these mechanisms, is the use of antioxidant enzyme arsenal, including CAT, POX, GST, APX, and SOD regulating the concentration of stressors, cellular superoxide (O^2−^) and hydrogen peroxide (H_2_O_2_), which upon uptake of heavy metals limit the production of OH radicals^4^. Different heavy metal effect the plants by versatile ways. As discussed earlier, some directly influence production of ROS, while others interfere with antioxidant defence mechanism^24^, hence a different enzymatic profile against each metal should be expected. In current study, exposure of *Petunia hybrida* L. to the selected HMs resulted in significantly higher antioxidant enzyme activity, which was enhanced with the increasing concentrations of HMs, and co-addition of EDTA along with HMs (Table 2). Similar findings were reported by other studies^4,12^. Moderate to high positive correlations were noted between all enzyme activities against selected HMs, except for APX with Cd (Supplementary Table S3). The increase in enzyme activates indicated stress induced by the HMs, as there were no significant variations in enzyme activities among the controls (with and without EDTA)^17^. Specific responses of anti-oxidant enzymes against a particular HM play an important role in metal toxicity using cellular defence strategy^25,26^. In this study, similar pattern was observed (Fig. 5) that the enzymatic profiles of *P. hybrida* L. for each of the HMs were different. A different mix of antioxidant enzyme CPC was noted for each HM, except in case of Cu, where APX activity was found having a very high CPC (70%). In another study, Wang, *et al*.^27^ found the same pattern of enzyme activity. They suggested that higher accumulation of HM (Cd) in non-accumulator ornamental plants, including African marigold (*Tagetes erecta*), scarlet sage (*Salvia splendens*) and sweet hibiscus (*Abelmoschus manihot*), resulted in such an effect.

### Heavy metal uptake and compartmentalization by *P. hybrida* L

The HM uptake was found to be dose dependent, and further enhanced by the addition of EDTA in the studied three compartments of *P. hybrida* L., comparable results were noted by Chen and Cutright^3^ and Kanwal, *et al*.^4^, while the HMs compartmentalization with in *P. hybrida* L. was found to vary for each of HMs (Fig. 6). With Cr, Ni, and Pb, significantly higher compartmentalization occurred in *P. hybrida* L. leaf. Similar outcome was achieved with Cd, but the distribution of Cd was comparatively homogenous than the other three. This represented that translocate HMs to above ground parts *P. hybrida* L. can lead to reduced plant vigour and aesthetics, as observed in this work that enhanced HM uptake occurred with facilitation of EDTA but with negative impacts on plant growth. For Cu, significantly higher levels were noted in roots, when *P. hybrida* L. was exposed to Cu stress, which increased with increasing the Cu in root and concentration in the external solution. Upon addition of EDTA the levels of Cu were increased in roots as well as in stem. This was due to formation of stable Cu-EDTA complex that are transported into shoot, and higher concentration end up there as compared to the treatment with no EDTA addition and Cu stress^28^. Other explanation to this variable metal compartmentalization was due to differential expression of proteins responsible for metal transporting with in different part of plant. These protein families included copper transporters (CTR or COPT)^29^, Cation Diffusion Facilitator (CDF)^30^, ZRT/IRT-like Protein (ZIP)^31^, Cation Exchanger (CAX)^32^, Natural Resistance-Associated Macrophage Protein (NRAMP)^30^, and Heavy Metal ATPase (HMA)^33^.

## Conclusion

The hydroponics system can provide an exact picture of the plant tolerance level for heavy metals due to non-interference of soil properties, micro/macro organisms and presence of other contaminating agents. We conclude that *P. hybrida* L. accumulated elevated levels of Cu, Cd, Ni and Pb in above ground parts, but this led to reduction in plant’s aesthetics, and reduced plant vigour. *P. hybrida* L. reduced Cu mobility to aerial plant parts and maintained higher concentration in the roots. The enzyme activates were increased, and this effect was dose dependent of HM concentrations while physiological and other biochemical parameters were influenced negatively by HMs uptake. The presence of chelating agent i.e. EDTA showed deleterious effects on plant health due to increased metal uptake, leading to physiological and biochemical stress on *P. hybrida*. To use *P. hybrida* L. in phytoremediation, the phytoextraction is not a good option. The use of *P. hybrida* L. with an appropriate combination of soil conditioners and plant growth promoting bacteria for other phytoremediation techniques, like phytostabilization is an unexplored area of research and may be feasible. Further, it is also important to investigate other ornamental plants, to know their metals/contaminants tolerance potential. Flowering plant belonging to *Catharanthus*, *Celosia*, *Cosmos, Dahlia, Mirabilis*, and *Nicotiana* genus are the good candidates for investigation due to wide cultivation, established agronomic practices, and higher biomass.

## Materials and Methods

### Plant material, experimental conditions and harvesting

Seeds of *Petunia hybrida* L. were sown in pots containing 3:1 mixture of soil to sand, and watered (when needed) with half strength Hoagland’s nutrient solution (pH 5.6) with the help of spray. Composition of full strength Hoagland’s nutrient solution was: 5 mM KNO_3_, 5 mM Ca(NO_3_)_2_.4H_2_O, 0.06 µM Fe(Na)EDTA, 2 mM MgSO_4_.7H_2_O, 1 mM NH_4_NO_3_, 46 µM H_3_BO_3_, 9 µM MnCl_2_.4H_2_O, 0.76 mM ZnSO_4_.7H_2_O, 0.204 mM CuSO_4_.5H_2_O, 0.450 mM Na_2_MoO_4_.2H_2_O, 0.5 mM KH_2_PO_4_, pH 5.6 (maintained with 1 M KOH or H_2_SO_4_). Pots were kept in dark till the seeds started to germinate, at 30 ± 1 °C in the day time and 24 ± 1 °C at night with a photoperiod of 16:8 (day:night), similar condition were maintained throughout the experiment. Upon seed germination, seedlings were transferred to wire house and after two weeks, equal sized uniform seedlings were carefully wrapped with filter wool between root and shoot, were held on thermophore sheet and were transferred to plastic containers containing full strength Hoagland’s nutrient solution (pH 5.6), lined with polythene sheet. The plants were acclimatized for 2 weeks, along with refreshing of Hoagland’s nutrient media and polythene sheet lining weekly, to prevent media contamination and depletion of nutrients.

After the acclimation, *Petunia hybrida* L. plants were treated for three weeks with HMs and EDTA as T1: Blank Control, T2: EDTA control (2.5 mM), T3: HM (50 μM), T4: HM (100 μM), T5: HM (50 μM) + EDTA (2.5 mM), and T6: HM (100 μM) + EDTA (2.5 mM). It is to be noted that all treatments were conducted in biological triplicates, T3, T4, T5, and T6 were different for each HM. Salts used for making representative HMs stocks were, cadmium chloride dihydrate (CdCl_2_.2H_2_O) for Cd, chromium nitrate (Cr(NO_3_)_2_) for Cr, copper sulphate pentahydrate (CuSO_4_.5H_2_O) for Cu, nickel chloride (NiCl_2_) for Ni, and lead nitrate (Pb(NO_3_)_2_) for Pb. After 3 weeks, plants were harvested, roots surface were washed with distilled water, and were used for plant physiological and metal uptake analysis. Samples for biochemical analysis were stored at −80 °C (for enzyme activities only) to avoid any disturbance in the activity. Each analysis was performed in biological triplicates.

### Plant physiological parameters

Plant physiological parameters, including plant height, root length, total number of leaf per plant, fresh and dried weight (FW and DW) of root, shoot and leaves were recorded using standard method as done by Arshad, *et al*.^34^ and Habiba, *et al*.^12^. Leaf area was calculated using ImageJ software^35^. Samples were dried at 60 °C till the constant weight and dried samples were used for acid digestion and then for atomic absorption spectrometry (AAS), with the help of Perkin Elmer, AAS-700, analysis for metal comparison in different plant compartments.

### Plant biochemical characters

Among plant biochemical characters of plant, stress injury and antioxidant enzyme activities were noted. For stress injury chlorophyll a, b, total chlorophyll and carotenoid content were quantified by using method described by Arnon^36^ and values were expressed in mg of chlorophyll g^−1^ of FW. Lipid peroxidation was noted in terms of malondialdehyde (MDA) by method adopted by Venkatachalam, *et al*.^37^, and was expressed in µM of MDA g^−1^ of FW. Electrolyte leakage (EL) was determined by the method as describe by Nishiyama, *et al*.^38^ and was expressed in percentage. H_2_O_2_ contents were determined according to Habiba, *et al*.^12^. Absorbance was taken at 410 nm and extinction coefficient of 0.28 µM^−1^ cm^−1^ was used for calculating H_2_O_2_ contents. The values were expressed in µM of H_2_O_2_ g^−1^ of FW. Quantification of plant enzyme and H_2_O_2_ content was done by preparation of plant extract. It was prepared using 100 mg of leaf’s tissue, homogenised with the help of pre-chilled mortar and pestle in 1 ml of potassium phosphate buffer (50 mM, pH 7.4 containing 0.5 mM EDTA). Resulted extracts were collected in 2 ml tube and were centrifuged at 10000 g for 15 min at 4 °C. After centrifugation, supernatant was carefully collected in 1.5 ml tube and was used and stored at 4 °C to prevent the deterioration^37^. Superoxide dismutase (SOD) activity was assayed by measuring its ability to inhibit the photochemical reduction of NBT using the method of Dhindsa, *et al*.^39^, by noting the absorbance at 560 nm of the reaction mixture. One unit of SOD activity was determined as the quantity of enzyme that induced 50% prohibition of photochemical reduction of the NBT. Catalase (CAT) activity was assayed by measuring the rate of disappearance of H_2_O_2_ in reaction mixture using the method of Maehly^40^. The decrease in H_2_O_2_ was followed as a decline in absorbance at 240 nm after 1 min (ε = 39.4 mM^−1^ cm^−1^). Ascorbate peroxidase (APX) activity was determined according to the method of Chen and Asada^41^, with minor modification. The oxidation of ascorbate was followed by the decrease in the absorbance at 240 nm (ε = 2.8 mM^−1^ cm^−1^). Guaiacol peroxidase (POX) activity was determined according to Upadhyaya, *et al*.^42^, and the activity was computed using the extinction coefficient of 26.6 mM^−1^ cm^−1^. Glutation-s-Transferase (GST) activity was assayed spectrophotometrically by measuring change of A_340_^43^. Reactions were initiated by the addition of 1-chloro-2,4-dinitrobenzene (CDNB), and A_340_ was monitored for 120 s in model of time-driver and values were computed using extinction coefficient of CDNB-glutathione conjugate (ε = 9.6 mM^−1^ cm^−1^). Values are expressed in Units g^−1^ of FW of sample for all enzyme activities, except for GST which was expressed in μM min^−1^ g^−1^ of FW.

### Statistical analysis

One-way ANVOA between individual metal treatment and among all metals was performed using SPSS, followed by Duncan’s multiple range test on each studied parameter. To find the correlation between metal uptake and studied parameters, Pearson’s correlation analysis was conducted, after testing the normality of data using the Shapiro-Wilk normality test. Further to find the relation of studied parameters with cumulative metal uptake stepwise multiple linear regressions (MLR) was employed. Data was min-max normalized prior to MLR, for the prevention of large numeric ranges dominating those with small numeric range, to reduce the potential bias into the data values exactly. In this normalization method, the recorded case value is subtracted with the minimum value of recorded in that case from each value of the attribute and followed by dividing the difference by the range of the studied case.

$$Z=\frac{x-\,{\rm{\min }}(x)}{{\rm{\max }}(x)-\,{\rm{\min }}(x)}$$

Where, *Z* is the normalized observed value of x, min and max are the minimum and maximum values in x given its range. The normalized values lay in the range [0, 1]. The advantage of this normalization is that it preserves all relationships of the data. Using MLR, multivariate model was constructed for metal uptake, the dependent variable Y, based on consciously selected studied variables (X). MLR coefficient (*R*^2^) showing highest value is ideal for the best equation. Based on these assumptions we can say:

$$Y={b}_{0}+{b}_{1}{X}_{1}+{b}_{2}{X}_{2}+\ldots +{b}_{nth}{X}_{nth}$$

Where, *Y* is the metal uptake (dependent variable), *X*_1_, *X*_2_, …, *X*_*n*_ are descriptive studied variables for plant physiology, enzyme activity, biochemical characteristic, and induced stress (independent variables), b_0_ is the constant, where the regression line intercepts the Y axis; b_ith_ (1 ≤ i ≤ nth) is the standard partial regression coefficient, representing the amount, the response variable Y changes when the descriptive studied variables changes 1 unit. This represents a model of the system under study, which can be used to investigate which variables influence its response and at what extent, and/or to predict the value of one variable when the others are known. The coefficient of regression (R^2^), had reliable competence between the predicted and measured values, and a higher R^2^, more than 0.75, is considered a good indicator of good fit model for stepwise MLR for cumulative metal uptake and studied parameters. Cumulative percentage contributions (CPC) of studied parameter with cumulative metal uptake for each metal was calculated using following equation;

$$Percentage\,contribution\,of\,specific\,parameter,\,i=(\frac{Bi}{\sum Bi})\times 100$$

where, B_i_ = MLR coefficient for specific parameter and ∑ B_i_ = sum MLR coefficient of all parameters^42,43^.

## Supplementary information

Supplementary Information
