# Supplementary material for: Combined application of selected heavy metals and EDTA reduced the growth of Petunia hybrida L
Source: Sci Rep. 2019 Mar 11;9:4138. doi: 10.1038/s41598-019-40540-7 (PMC6411725; doi:10.1038/s41598-019-40540-7)
Supplement: Supplementary file 1 — Supplementary Information [file 41598_2019_40540_MOESM1_ESM.docx]

**Supplementary Information**

**Combined application of selected heavy metals and EDTA reduced the growth of *Petunia hybrida* L.**

Aqib Hassan Ali Khan^1^, Tayyab Ashfaq Butt^2^, Cyrus Raza Mirza^2^, Sohail Yousaf^1^, Ismat Nawaz^3^, Mazhar Iqbal^1*^

*^1^Department of Environmental Sciences, Faculty of Biological Sciences, Quaid-i-Azam University,* 45320, *Islamabad, Pakistan*

*^2^Department of Architectural Engineering, College of Engineering, University of Hail, Hail, Saudi Arabia*

*^3^Department of Environmental Sciences, COMSATS University Islamabad, Abbottabad Campus, Abbottabad, Pakistan*

*Correspondence

Dr. Mazhar Iqbal (Ph.D.)

Department of Environmental Sciences

Faculty of Biological Sciences

Quaid-i-Azam University

45320 – Islamabad, Pakistan

E-mail: [miqbal@qau.edu.pk](mailto:miqbal@qau.edu.pk)

Phone: +923116691956

**Contents**

**Supplementary Table S1.** Pearson’s correlation between selected HMs and *P. hybrida* L physiological parameters

**Supplementary Table S2.** Pearson’s correlation between selected HMs and *P. hybrida* L. studied stress indicating parameters

**Supplementary Table S3.** Pearson’s correlation between selected HMs and *P. hybrida* L. studied enzyme activities

**Supplementary Table S1.** Pearson’s correlation between selected HMs and *P. hybrida* L physiological parameters

|  | *Leaf* | | | |  | *Root* | | |  | *Shoot* | | |
| --- | --- | --- | --- | --- | --- | --- | --- | --- | --- | --- | --- | --- |
|  | Plant^-1^ (n) | Area (cm^2^) | Fresh weight (g) | Dried weight (g) |  | Length (cm) | Fresh weight (g) | Dried weight (g) |  | Length (cm) | Fresh weight (g) | Dried weight (g) |
| *Cd* | -0.935 | -0.708 | -0.717 | -0.715 |  | -0.786 | -0.840 | -0.657 |  | -0.819 | -0.837 | -0.871 |
| *Cr* | -0.724 | -0.703 | -0.681 | -0.558 |  | -0.750 | -0.796 | -0.706 |  | -0.746 | -0.738 | -0.789 |
| *Cu* | -0.948 | -0.712 | -0.757 | -0.648 |  | -0.831 | -0.856 | -0.755 |  | -0.790 | -0.852 | -0.874 |
| *Ni* | -0.838 | -0.488* | -0.455* | -0.507* |  | -0.788 | -0.845 | -0.734 |  | -0.666 | -0.828 | -0.869 |
| *Pb* | -0.704 | -0.431* | -0.481* | -0.524* |  | -0.663 | -0.799 | -0.722 |  | -0.660 | -0.792 | -0.839 |
| Pearson’s correlation coefficients were significant at the 0.005 probability level, except * which are significant at 0.05, n = 18. | | | | | | | | | | | | |

**Supplementary Table S2.** Pearson’s correlation between selected HMs and *P. hybrida* L. studied stress indicating parameters

|  | *Stress Indicators* | | | | | | |
| --- | --- | --- | --- | --- | --- | --- | --- |
|  | MDA content (µM g^-1^ FW) | Electrolyte Leakage (%) | H_2_O_2_ content (µM g^-1^ FW) | Chlorophyll a (mg g^-1^ FW) | Chlorophyll b (mg g^-1^ FW) | Total chlorophyll (mg g^-1^ FW) | Carotenoid (mg g^-1^ FW) |
| *Cd* | 0.878 | 0.855 | 0.771 | -0.705 | -0.672 | -0.709 | -0.589 |
| *Cr* | 0.761 | 0.765 | 0.618 | -0.382^+^ | -0.689 | -0.577 | -0.816 |
| *Cu* | 0.841 | 0.773 | 0.927 | -0.741 | -0.615 | -0.730 | -0.768 |
| *Ni* | 0.823 | 0.825 | 0.696 | -0.859 | -0.556 | -0.819 | -0.816 |
| *Pb* | 0.566* | 0.620 | 0.904 | -0.644 | -0.676 | -0.683 | -0.752 |
| Pearson’s correlation coefficients were significant at the 0.005 probability level, except * which are significant at 0.05,  n = 18, ^+^ represent insignificant correlation at 0.05 or 0.005 *P* value. | | | | | | | |

**Supplementary Table S3.** Pearson’s correlation between selected HMs and *P. hybrida* L. studied enzyme activities

|  | *Enzyme activities* | | | | |
| --- | --- | --- | --- | --- | --- |
|  | Catalase^1^ | Guaiacol peroxidase^1^ | Glutathione S-transferase^1^ | Ascorbate peroxidase^1^ | Superoxide dismutase^1^ |
| *Cd* | 0.640 | 0.613 | 0.633 | 0.194^+^ | 0.746 |
| *Cr* | 0.889 | 0.690 | 0.812 | 0.921 | 0.590 |
| *Cu* | 0.733 | 0.453* | 0.602 | 0.901 | 0.746 |
| *Ni* | 0.757 | 0.755 | 0.742 | 0.734 | 0.704 |
| *Pb* | 0.810 | 0.728 | 0.595 | 0.530* | 0.668 |
| \| Pearson’s correlation coefficients were significant at the 0.005 probability level, except * which are significant at 0.05, n = 18.  + represent insignificant correlation at 0.05 or 0.005 p value. \| \| --- \|   ^1^ all enzyme activities were expressed in U g^-1^ FW of plant, while for GST was in μmol min^-1^ g^-1^ FW of plant. | | | | | |
